# Catabolic regulation analysis of *Escherichia coli *and its *crp, mlc, mgsA, pgi *and *ptsG *mutants

**DOI:** 10.1186/1475-2859-10-67

**Published:** 2011-08-11

**Authors:** Ruilian Yao, Yuki Hirose, Dayanidhi Sarkar, Kenji Nakahigashi, Qin Ye, Kazuyuki Shimizu

**Affiliations:** 1 Department of Bioscience and Bioinformatics, Kyushu Institute of Technology, Iizuka, Fukuoka 820-8502, Japan; 2 State Key Laboratory of Bioreactor Engineering, East China University of Science and Technology, Shanghai 200237, China; 3 Institute of Advanced Bioscience, Keio University, Tsuruoka, Yamagata, 997-0017, Japan

## Abstract

**Background:**

Most bacteria can use various compounds as carbon sources. These carbon sources can be either co-metabolized or sequentially metabolized, where the latter phenomenon typically occurs as catabolite repression. From the practical application point of view of utilizing lignocellulose for the production of biofuels etc., it is strongly desirable to ferment all sugars obtained by hydrolysis from lignocellulosic materials, where simultaneous consumption of sugars would benefit the formation of bioproducts. However, most organisms consume glucose prior to consumption of other carbon sources, and exhibit diauxic growth. It has been shown by fermentation experiments that simultaneous consumption of sugars can be attained by *ptsG, mgsA *mutants etc., but its mechanism has not been well understood. It is strongly desirable to understand the mechanism of metabolic regulation for catabolite regulation to improve the performance of fermentation.

**Results:**

In order to make clear the catabolic regulation mechanism, several continuous cultures were conducted at different dilution rates of 0.2, 0.4, 0.6 and 0.7 h^-1 ^using wild type *Escherichia coli*. The result indicates that the transcript levels of global regulators such as *crp, cra, mlc *and *rpoS *decreased, while those of *fadR, iclR, soxR/S *increased as the dilution rate increased. These affected the metabolic pathway genes, which in turn affected fermentation result where the specific glucose uptake rate, the specific acetate formation rate, and the specific CO_2 _evolution rate (CER) were increased as the dilution rate was increased. This was confirmed by the ^13^C-flux analysis. In order to make clear the catabolite regulation, the effect of *crp *gene knockout (Δ*crp*) and crp enhancement (*crp^+^*) as well as *mlc, mgsA, pgi *and *ptsG *gene knockout on the metabolism was then investigated by the continuous culture at the dilution rate of 0.2 h^-1 ^and by some batch cultures. In the case of Δ*crp *(and also Δ*mlc*) mutant, TCA cycle and glyoxylate were repressed, which caused acetate accumulation. In the case of *crp^+ ^*mutant, glycolysis, TCA cycle, and gluconeogenesis were activated, and simultaneous consumption of multiple carbon sources can be attained, but the glucose consumption rate became less due to repression of *ptsG *and *ptsH *by the activation of Mlc. Simultaneous consumption of multiple carbon sources could be attained by *mgsA, pgi*, and *ptsG *mutants due to increase in *crp *as well as *cyaA*, while glucose consumption rate became lower.

**Conclusions:**

The transcriptional catabolite regulation mechanism was made clear for the wild type *E. coli*, and its *crp, mlc, ptsG, pgi, and mgsA *gene knockout mutants. The results indicate that catabolite repression can be relaxed and *crp *as well as *cyaA *can be increased by *crp^+^, mgsA, pgi*, and *ptsG *mutants, and thus simultaneous consumption of multiple carbon sources including glucose can be made, whereas the glucose uptake rate became lower as compared to wild type due to inactivation of *ptsG *in all the mutants considered.

## Background

It is quite important to understand how the culture environment affects the cell metabolism. Among the culture environments, carbon source is by far important in practice. In particular, carbon catabolite repression has been paid recent attention [1], where most bacteria selectively consume substrates from a mixture of different carbon sources, and exhibit diauxic growth. Namely, most organisms consume glucose prior to consumption of other carbon sources. From the application point of view of utilizing lignocellulose for the production of biofuels etc., it is strongly desirable to ferment all sugars obtained by hydrolysis from lignocellulosic materials simultaneously [2]. In particular, simultaneous consumption of sugars would benefit the formation of bioproducts. Several attempts have, therefore, been made in the past, where *ptsG *was mutated for ethanol production [3] and for lactate production [4] from a mixture of glucose and xylose. Noting that the glucose consumption rate becomes low by a *ptsG *mutation and *pts *mutation [5], the *gal *regulon genes, which encode non-PTS transporter, were enhanced [6,7]. More recently, *mgsA *gene knockout which encodes the initial enzyme from DHAP to methylglyoxal pathway was considered for the simultaneous consumption of multiple sugars [8], but it is not explained on its mechanisms. Moreover, it has been shown that cAMP increases for *pyk *knockout mutant [9], but this may not be a significant contribution for the simultaneous consumption of a mixture of sugars, since the increase in cAMP is limited. Yet, another idea of co-fermentation strategy has been proposed, where this process uses two substrate-selective strains of *E. coli*, one of which is unable to consume glucose and the one which is unable to consume xylose for lactate production [10]. However, it may be difficult to analyze the mixed culture, since one cannot discriminate two strains, and one population may washout during continuous culture.

In the present study, we attempted to clarify the catabolic regulation mechanism of *E. coli *based on fermentation characteristics and selected gene transcript levels. In order to understand the catabolic regulation, the recognition and adjustment mechanisms must be understood in view of the relationships between global regulators and the metabolic pathway genes. In the catabolic regulation, cAMP-Crp complex plays an important role. The center for this regulatory network is the phosphoenolpyruvate (PEP): carbohydrate phosphotransferase systems (PTSs). These systems are involved in both transport and phosphorylation of carbohydrates for the regulation of the main metabolic pathways. The PTS in *E. coli *consists of two cytoplasmic proteins such as EI (enzyme I) and HPr (histidine-containing protein), as well as carbohydrate-specific EII (enzyme II) complexes. The unphosphorylated EIIA^Glc ^inhibits the uptake of other non-PTS carbohydrates by a so-called inducer exclusion. Phosphorylated EIIA^Glc ^activates the adenylate cyclase (Cya) and leads to an increase in the intracellular cAMP level, which then combines with Crp forming cAMP-Crp complex, and controls certain metabolic pathway genes [11-13].

In order to increase understanding of the catabolic regulation phenomenon, several continuous cultures were conducted for wild type *E. coli *at different dilution rates in the present study. Moreover, the effects of *crp *gene knockout and *crp *enhancement (*crp*^+^) on the metabolic regulation were also investigated. Since Crp activates *ptsG*, while Mlc represses it, we also investigated the effect of *mlc *gene knockout in addition to *crp *knockout and enhancement on the metabolism. In the present investigation, we also considered *mgsA, pgi *and *ptsG *mutants as well to clarify the mechanism of catabolite repression. Finally, the effects of those mutations on the simultaneous consumption of a mixture of carbon sources such as glucose and xylose were investigated.

## Results

### Effect of dilution rate on the metabolic regulation in wild type *E. coli*

**Figure 1:**
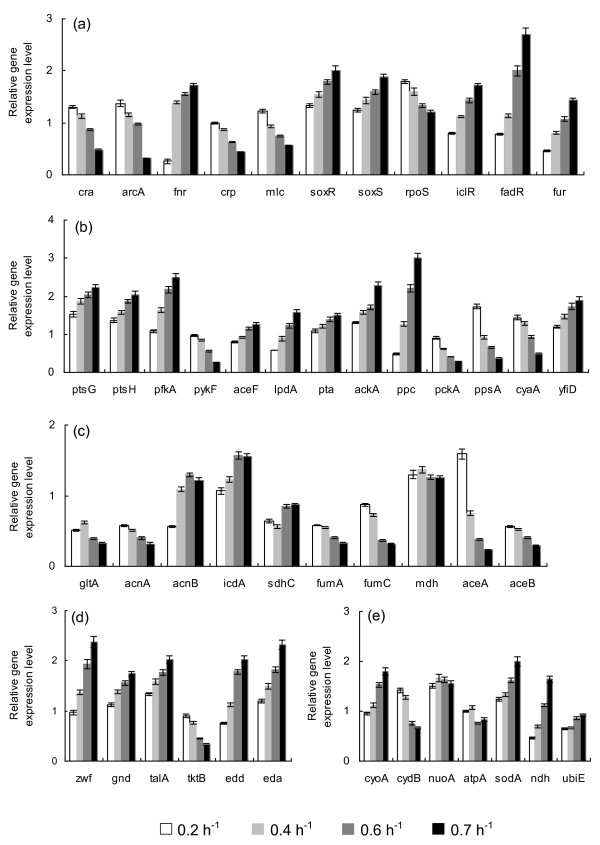
**The effect of dilution rate on the gene transcript levels of wild type *E. coli***. (a) Global regulator genes; (b) PTS, glycolysis, anaplerotic pathway, *cyaA *and *yfiD *genes; (c) TCA and glyoxylate pathway genes; (d) PP pathway genes; (e) Respiratory chain genes.

Table 1 shows the fermentation characteristics of the wild type *E. coli *for the continuous culture at different dilution rates, where it indicates that the specific glucose uptake rate, acetate production rate, and the specific CO_2 _evolution rate (CER) were increased as the dilution rate was increased. Figure 1 shows the effect of the dilution rate (the specific growth rate) on gene transcript levels, where it indicates that in accordance with the fermentation data of the increased specific glucose consumption rate, the transcript levels of *ptsG, ptsH*, and *pfkA *were increased as the dilution rate increased, where *cra *transcript level decreased and *crp *as well as *mlc *decreased accordingly (Additional file 1). The decrease in *crp *is also coincident with the decrease in *cyaA *which encodes Cya. The transcript levels of *zwf, gnd, edd*, and *eda *were increased as the dilution rate increased in accordance with the decrease in *cra*. The transcript level of *ppc *increased while *pckA *decreased as the dilution rate increased as evidenced by ^13^C-metabolic flux result (Additional file 2). Moreover, the transcript levels of *fadR *and *iclR *increased, and *aceA *and *aceB *decreased as the dilution rate increased. In accordance with the increase in the specific acetate production rate, the transcript levels of *pta *and *ackA *increased. TCA cycle genes such as *gltA, acnA, fumA, C *decreased, while *acnB, icdA *and *lpdA *increased (except the case of dilution rate at 0.7 h^-1^) as the dilution rate increased. In accordance with the increase in *soxR/S *transcript levels, *zwf *and *sodA *increased. In accordance with the decrease in the transcript level of *arcA*, the transcript level of *cyoA *increased, while *cydB *decreased as the dilution rate increased, where the latter phenomenon also coincided with the decrease in *cra *transcript level (Additional file 1). Further observation indicates that in accordance with the decrease in *rpoS *transcript level, *tktB, acnA, fumC *decreased, while *fur *increased as the dilution rate increased in accordance with the increase in *soxR/S*.

**Table 1:** Effect of the dilution rate on fermentation characteristics of wild type *E. coli*

Dilution rate (h^-1^)	Biomass conc. (g/L)	Glucose conc. (g/L)	Glucose uptake rates (mmol/g/h)	Acetate formation rates (mmol/g/h)	Biomass yield (g/g)	CER (mmol/g/h)	Carbon recovery
0.2	1.45 ± 0.06	ND*	3.07 ± 0.13	ND*	0.37 ± 0.015	9.15	94%
0.4	1.87 ± 0.09	ND*	4.75 ± 0.23	0.01	0.47 ± 0.023	11.61	98%
0.6	2.0 ± 0.09	ND*	6.67 ± 0.3	0.88 ± 0.04	0.5 ± 0.023	13.17	98%
0.7	1.93 ± 0.08	ND*	8.05 ± 0.34	1.33 ± 0.06	0.48 ± 0.02	15.83	97%

ND*: not detectable, where glucose detectable limit was 0.038 g/l.

Additional file 2 shows the comparison of the metabolic flux distributions of *E. coli *at the dilution rates of 0.2 h^-1^, 0.4 h^-1^, 0.6 h^-1 ^and 0.7 h^-1^, where it indicates that the metabolic pathway fluxes such as glycolysis, PP pathway, and anaplerotic pathway fluxes increased as the dilution rate increased, where those results are consistent with the gene transcript levels as stated above. Based on the flux result, the specific ATP production rate and the specific NADPH formation rate computed using Eqs. (1) and (2) as stated in Methods section are plotted with respect to the dilution rate in Additional file 3, which indicates that these are linearly correlated with the specific growth rate.

### Effect of *crp *gene mutation on the metabolism

**Figure 2:**
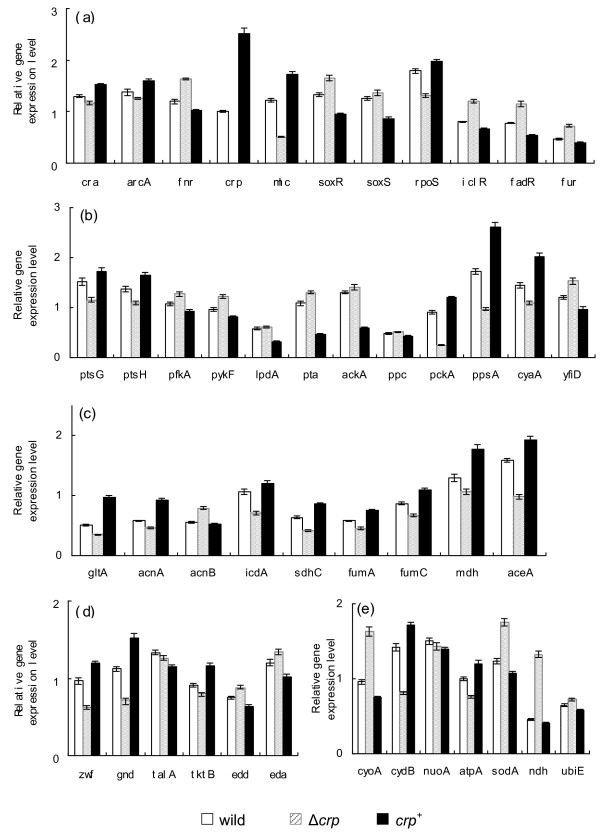
**Comparison of gene transcript levels of the wild type, *crp *knockout mutant, and *crp^+ ^*mutant**. (a) Global regulator genes; (b) PTS, glycolysis, anaplerotic pathway, *cyaA *and *yfiD *genes; (c) TCA and glyoxylate pathway genes; (d) PP pathway genes; (e) Respiratory chain genes. 1.

Table 2 shows the effect of *crp *gene knockout and *crp *enhancement (*crp*^+^) on the fermentation characteristics at the dilution rate of 0.2 h^-1^, where it indicates that the specific glucose uptake rate was lower and the cell concentration was higher as compared to the wild type, and the specific acetate production rate was higher for *crp *knockout mutant. In the case of *crp*^+ ^mutant, the fermentation characteristics were similar to the wild type by considering the statistical significance. Figure 2a shows the transcript levels of *crp *knockout mutant and *crp*^+ ^mutant as compared to wild type, where it indicates that *mlc *was down-regulated (P < 0.01) in *crp *knockout mutant, and up-regulated (P < 0.01) in *crp*^+ ^mutant. Figure 2b indicates that *ptsG *and *ptsH *transcript levels changed in a similar fashion as *mlc *and *crp*, which corresponds to the lower glucose uptake rate for *crp *knockout mutant and similar to the wild type for *crp*^+ ^mutant (Table 2). Figure 2d also indicates that *acnA, gltA, fumA, mdh, pckA, sdhC *all changed in a similar fashion as *crp *(Additional file 1). The *cydB *transcript level changed in a similar fashion as *arcA*, while *cyoA *gene changed in a reverse fashion (Additional file 1). The *sodA *and *fur *changed in a similar fashion as *soxR/S*. The *tktB *and *fumC *changed in a similar fashion as *rpoS *(Additional file 1). The changing patterns of *aceA, icdA *and *pckA *transcript levels were similar as *cra*, while reverse patterns may be seen for *fadR *and *iclR*.

**Table 2:** Effect of the specific gene mutation on the fermentation characteristics at the dilution rate of 0.2 h^-1^

Strains	Biomass conc. (g/L)	Glucose conc. (g/L)	Glucose uptake rate (mmol/g/h)	Acetate formation rate (mmol/g/h)	Biomass yield (g/g)
BW25113	1.45 ± 0.06	ND*	3.07 ± 0.13	ND*	0.37 ± 0.015
Δ*crp *	1.57 ± 0.07	ND*	2.83 ± 0.13	0.35 ± 0.018	0.39 ± 0.018
*crp^+ ^*	1.42 ± 0.07	ND*	3.12 ± 0.14	ND*	0.36 ± 0.018
Δ*pgi*	1.6 ± 0.08	ND*	2.77 ± 0.14	ND*	0.4 ± 0.021
Δ*mlc*	1.65 ± 0.08	ND*	2.69 ± 0.13	ND*	0.41 ± 0.019
Δ*mgsA*	1.55 ± 0.07	ND*	2.87 ± 0.14	0.1 ± 0.005	0.39 ± 0.018

ND*: not detectable, where glucose detectable limit was 0.038 g/l.

### Effect of *mlc *gene knockout on the metabolism

**Figure 3:**
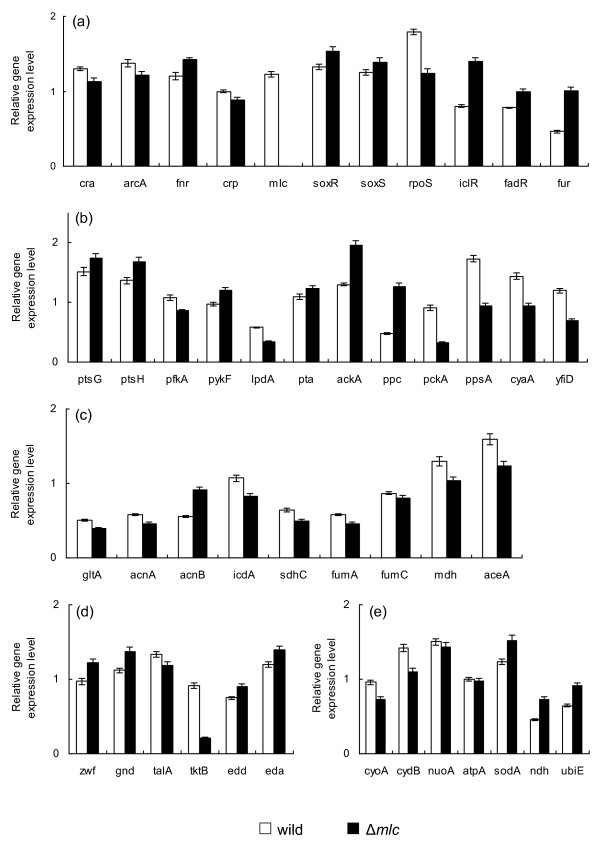
**Comparison of gene transcript levels of the wild type and *mlc *knockout mutant**. (a) Global regulator genes; (b) PTS, glycolysis, anaplerotic pathway, *cyaA *and *yfiD *genes; (c) TCA and glyoxylate pathway genes; (d) PP pathway genes; (e) Respiratory chain genes.

Table 2 also shows the fermentation characteristics of *mlc *mutant at the dilution rate of 0.2 h^-1^, where it indicates that the specific glucose uptake rate was lower than wild type. Figure 3 shows the transcript levels of *mlc *mutant as compared to those of the wild type, where the transcript level of *crp *decreased (P < 0.05) as compared to the wild type. Due to *mlc *gene knockout, the transcript levels of *ptsG *and *ptsH *increased (P < 0.05 and P < 0.01, respectively), which may be also coincided with the decrease in *cra *transcript level (P < 0.01). However, this does not cause the specific glucose consumption rate to increase, which may be partly due to decrease in *pfkA *gene transcript level (P < 0.01), while *pykF *increased (P < 0.01). The transcript level of *rpoS *decreased (P < 0.01) and *tktB, fumC *were down-regulated (P < 0.01 and P < 0.05, respectively) accordingly (Additional file 1). The transcript levels of *soxR/S *increased (P < 0.01 and P < 0.05, respectively), and *sodA *increased (P < 0.01) as compared to the wild type.

### Effect of *mgsA and pgi *gene knockout on the metabolism

**Figure 4:**
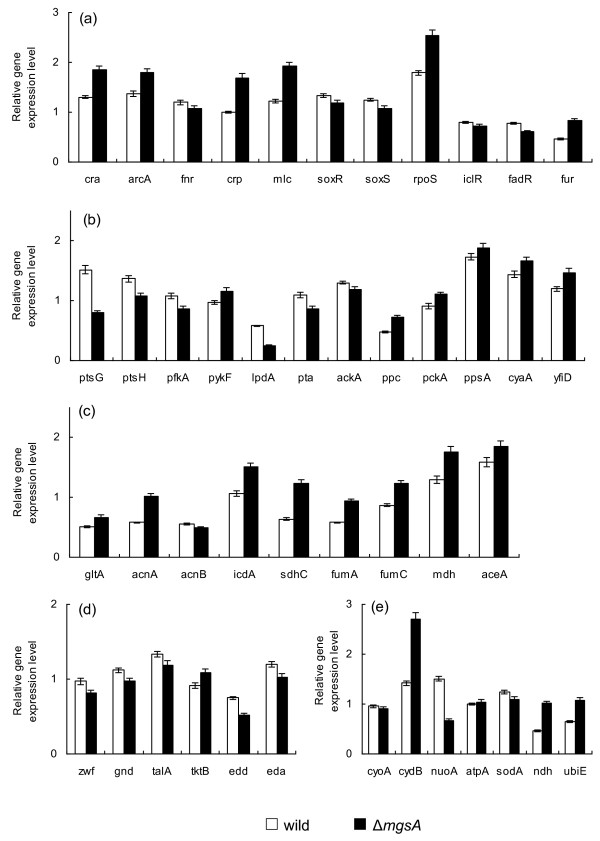
**Comparison of gene transcript levels of the wild type and *mgsA *knockout mutant**. (a) Global regulator genes; (b) PTS, glycolysis, anaplerotic pathway, *cyaA *and *yfiD *genes; (c) TCA and glyoxylate pathway genes; (d) PP pathway genes; (e) Respiratory chain genes.

Table 2 also shows the effects of *mgsA *and *pgi *gene knockout on the fermentation characteristics, where it indicates that the glucose consumption rate decreased, and the cell yield increased. Figure 4 shows the transcript levels of *mgsA *mutant as compared to wild type, where it indicates that the transcript levels of *cyaA *and *crp *as well as *mlc *increased (P < 0.05, P < 0.01 and P < 0.01, respectively). The *cra *transcript level increased (P < 0.01) and thus the transcript levels of *ptsG, ptsH *and *pfkA *decreased (P < 0.01 for all genes), which is consistent with the decreased glucose consumption rate, while *pckA, ppsA *and *aceA *were all increased (P < 0.01, P < 0.05, P < 0.05, respectively). The increase in *aceA *transcript level (P < 0.05) coincided with the decrease in *fadR *and *iclR *transcript levels (P < 0.05 and P < 0.01, respectively).

### Fermentation characteristics of using multiple carbon sources

The modulation of catabolite regulation may be applied for the simultaneous consumption of multiple carbon sources originated from the hydrolysis of lignocellulose. Additional file 4a shows the batch cultivation result for wild type where the mixture of glucose and xylose was used as a carbon source, where it indicates that glucose was first consumed, while xylose was consumed after glucose was depleted due to catabolite repression in the wild type. Additional file 4b shows the comparison of batch cultures of wild type and *crp*^+ ^mutant, where it indicates that although simultaneous consumption of both sugars could be attained, the substrate uptake rate became lower, which may be due to repression of *pts *by increased Mlc caused by the increased *crp *gene transcript level. Additional file 5 shows the comparison of wild type and its *crp*^+ ^mutant for the continuous culture, which indicates that xylose tends to accumulate as the dilution rate increases for wild type (Additional file 5a) while both glucose and xylose concentrations are similar for *crp*^+ ^mutant (Additional file 5b). The decreased glucose consumption rate for *crp*^+ ^mutant as observed in the batch culture may have caused lower cell and acetate concentrations in the continuous culture (Additional file 5). Additional file 6 shows the continuous culture of *ptsG *mutant for the case of using a mixture of glucose and xylose. Although simultaneous consumption of both sugars can be attained at lower dilution, the glucose consumption rate became lower, and glucose concentration increased at higher dilution rate for *ptsG *mutant as illustrated by additional file 6. The similar phenomenon was observed for *pgi *mutant, where glucose consumption rate became lower (data not shown).

## Discussion

The effect of dilution rate (the specific growth rate) on gene transcript levels (Figure 1) and fermentation characteristics (Table 1) may be explained as follows: As the dilution rate increased, the glucose concentration increased (though not detectable in Table 1), which caused *cra *transcript level to be decreased (Figure 1a). The *crp *transcript level also decreased as the dilution rate was increased, which may be due to decrease in phosphorylated EIIA^Glc^, caused by higher glucose concentration. This deactivated Cya (as implied by *cyaA *in Figure 1b), which in turn caused decrease in cAMP concentration, and thus cAMP-Crp or *crp *transcript level decreased (Figure 1a). Note that cAMP level starts to increase when glucose concentration becomes less than about 0.3 mM [14]. The increases in *fadR *and *iclR *(Figure 1a) also coincided with the increase in the glucose concentration as the dilution rate was increased. The decrease of *rpoS *(Figure 1a) may be also explained as less nutrient stress as the dilution rate was increased. The decrease of *arcA *transcript level and increases of *soxR/S *transcript levels may be due to higher dissolved oxygen concentration caused by lower cell concentration at higher dilution rate (Table 1).

**Figure 5:**
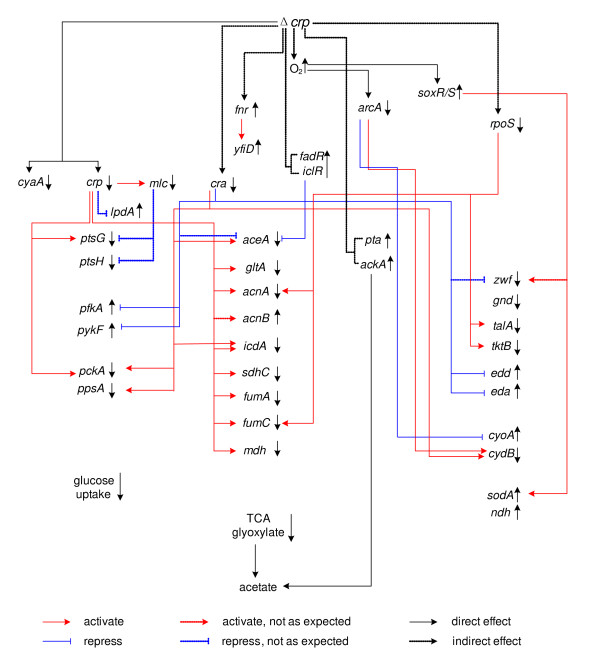
**The overall regulation of Δ*crp *mutant**.

Referring to Figure 5 and Additional file 1, some of the metabolic pathway transcript levels may be explained with respect to the change in global regulators. The decreases of *aceA *and *aceB *transcript levels coincided with the increase of those of *iclR *and *fadR *as the dilution rate was increased. This also corresponds to the flux result (Additional file 2). The decreases of the transcript levels of *aceA, B*, and *pckA, ppsA, acnA *(Figure 1 b, c) coincide with the decrease in the *cra *transcript level (Figure 1a), which also caused increase in *eda, edd, pfkA, ptsH *transcript levels. The increase of *cyoA *and decrease of *cydB *coincide with the decrease in *arcA *as the dilution rate was increased, where the decrease in *cydB *also coincides with the decrease in *cra*. Note that *ppc *transcript level increased as the dilution rate increased as confirmed by the flux result (Additional file 2), which may be due to increased F1,6BP. Although *lpdA *and *aceF *transcript levels increased as the dilution rate was increased, TCA cycle gene transcript levels such as *gltA, acnA, fumA, fumC *tended to decrease, while *acnB *and *icdA *(*sdhC*) transcript levels tended to increase. The increase in *lpdA *transcript level may be due to decrease in *crp*, where cAMP-Crp repressed such gene expression. The decreases of *acnA *and *fumC *transcript levels may be caused by the decrease in *rpoS *(Additional file 1). It has been shown that *acnB *is dominant for the exponential growth phase, while *acnA *is dominant at the stationary phase [15,16], which indicates that the continuous culture at higher dilution rate mimics the cell growth phase in the batch culture, while it mimics the stationary phase at low dilution rate. The increases in *soxR/S *caused *zwf *and *sodA *to be increased, whereas *acnA *and *fumC *decreased. The latter may be due to decrease in *rpoS *transcript level (Additional file 1). The increase in *yfiD *may be due to increase in *fnr *transcript level, though Fnr may be inactive form under aerobic condition. Upon complex formation of cAMP-Crp, it activates genes encoding the glucose PTS system, the TCA cycle and gluconeogenesis (Additional file 1). Although *crp *transcript level decreased as the dilution rate was increased (Figure 1a) as stated above, *ptsG *and *ptsH *transcript level increased (Figure 1b). This may be caused by the decrease in *cra*, where *pfkA *also increased.

As stated above in the result section, the specific glucose consumption rate decreased in the *crp *knockout mutant, as compared to the wild type, being almost the same in *crp^+ ^*mutant. The *ptsG *gene is activated by Crp, but also repressed by Mlc. Note that, the changing pattern of *crp *transcript level (Figure 2a) coincides with that of *mlc *(Figure 2a) as well as *cyaA*. It has been reported that there is a Crp binding region in the promoter region of *mlc *gene [17].

In the case of *crp *knockout mutant, the decrease in glucose consumption rate may be caused by down regulations of *ptsG *and *ptsH *(P < 0.01 and P < 0.01, respectively), whereas *pfkA *and *pykF *were up-regulated (P < 0.01 and P < 0.01, respectively). The former may be caused by *crp *knockout, while the latter may be due to down-regulation of *cra *(P < 0.01) (Figure 2a), where *mlc *might not be dominant. The *crp *knockout caused TCA cycle genes such as *gltA, acnA, sdhC, fumA*, and *mdh *to be down regulated (P < 0.01, P < 0.01, P < 0.01, P < 0.05 and P < 0.01, respectively). Figure 2a shows *cra *to be down-regulated (P < 0.01), which may be caused by higher glucose concentration, though it is less than detectable level. The down regulation of *cra *(P < 0.01) also caused *icdA *gene as well as gluconegogenic pathway genes *pckA *and *ppsA *to be down regulated (P < 0.01, P < 0.05 and P < 0.01, respectively) (Figure 2a, b, c). The decrease in glyoxylate pathway gene *aceA *(P < 0.01) (Figure 2c) may be caused by the increases of *fadR *and *iclR *(P < 0.01 and P < 0.01, respectively), which may be due to higher glucose concentration. The decreased activities of TCA cycle and glyoxylate pathway may have caused acetate to accumulate, where this is also reflected by the up-regulation of *pta *and *ackA *transcript levels (P < 0.01 and P < 0.05, respectively) (Figure 2b). The batch culture of Δ*crp *mutant indicates that the acetate formed during cell growth phase, could not be consumed during stationary phase (Additional file 7). The decreased transcript levels of *acnA, fumC*, and *tktB *(P < 0.01, P < 0.01 and P < 0.01, respectively) may also be due to down regulation of *rpoS *(P < 0.01), which may be due to less nutrient stress caused by the increase of glucose concentration. The *sodA *gene transcript level increased (P < 0.01) (Figure 2e), which is consistent with up-regulation of *soxR/S *(P < 0.01 and P < 0.05, respectively) (Figure 2a), which might be due to increase in oxygen concentration. The decrease of *arcA *(P < 0.05) (Figure 2a) is also reflected by this hypothesis, whereas its effect on TCA cycle genes may be minor and not dominant. The overall regulation mechanism is illustrated in Figure 5.

In the case of *crp^+ ^*mutant, the *ptsG *and *ptsH *transcript levels were increased (P < 0.05 and P < 0.01, respectively), whereas *pfkA *and *pykF *were decreased (P < 0.01 and P < 0.01, respectively). It should be noted that *crp *gene enhancement caused increase in *mlc *gene (P < 0.01) (Figure 2a), and thus Mlc might have repressed *ptsG *and *ptsH*. This seems to be dependent on the culture condition. This might happen when glucose concentration is higher as will be shown for the batch culture. Different from the case of Δ*crp *mutant, TCA cycle genes and glyoxyalte pathway gene as well as gluconeogenic pathway genes were up-regulated. The activation of TCA cycle may have caused the decrease in cell yield.

In the case of *mlc *mutant (Figure 3), *crp *as well as *cyaA *transcript levels were also decreased (P < 0.05 and P < 0.01, respectively). The decreases of *crp *(P < 0.05) and *cra *(P < 0.01) both caused glucose uptake and glycolysis pathway genes (except *pfkA*) to be up-regulated, while gluconeogensis, TCA cycle and glyoxylate pathway genes were decreased. Although not reflected in the continuous culture (Table 2), the above phenomenon may cause more acetate formation as also implied by the up-regulations of *pta *and *ackA *genes (P < 0.05 and P < 0.01, respectively) (Figure 3b). In fact, this happened in the batch culture, and the acetate formed during the cell growth phase could not be consumed during the stationary phase (Additional file 8).

In the case of *mgsA *mutant (Figure 4), PTS may be repressed as shown by the down regulations of *ptsG *and *ptsH *as well as *pfkA *transcript level (P < 0.01 for all genes), which may be partly due to accumulation of G6P caused by *mgsA *gene knockout. A similar phenomenon may be seen also for *pgi *and *pfk *gene knockout (data not shown). The increase in G6P may cause mRNA of *ptsG *to be unstable [18], and the phosphorylation of glucose at EIICB by EIIA^Glc^-P might be disrupted, and this have caused *crp *(and therefore *mlc*) transcript level as well as *cyaA *and *cra *to be up-regulated (P < 0.01, (P < 0.01), P < 0.05 and P < 0.01, respectively), which in turn reduced the acetate formation as implied by the down-regulations of *pta *and *ackA *transcript levels (P < 0.01 and P < 0.05, respectively). The increases in *cydB *(P < 0.01) may be due to the increased transcript levels of *cra *and *arcA *(P < 0.01 and P < 0.01, respectively). The transcript level of *rpoS *is also increased (P < 0.01) for *mgsA *mutant, which might be due to stress imposed on catabolism.

The fermentation results of using a mixture of glucose and xylose indicate that simultaneous consumption of multiple carbon sources can be attatined by *crp^+^, mgsA pgi *(*pfkA*), and *ptsG *mutants. The mechanism for this phenomenon is due to increase in *crp *transcript level, which may be explained as the deficient *ptsG *expression for *mgsA, pgi *(*pfkA*) and *ptsG *mutants. This means that the glucose uptake rate may be decreased due to less activity of EIICB^Glc ^encoded by *ptsG*, while Mlc represses the *ptsG *and *ptsH *for the case of *crp^+ ^*mutant. In the case of deficient EIICB^Glc^, glucose transport may be made by GalP and MglBAC, and it is phosphorylated by ATP and converted to G6P, but this process may be less efficient than PTS. The increase in *crp *transcript level for those mutants may have caused TCA cycle as well as glyoxylate pathway to be activated, and thus less acetate may be formed.

In the case of *pgi *mutant, the glucose consumption rate became low, resulting in the significantly low cell growth rate [19,20]. The *mgsA *mutant may have caused G6P to be increased but its extent may be low as compared to *pgi *(*pfkA*) mutant, and thus the glucose uptake rate may retain higher but less than wild type. Although *pyk *mutant may cause G6P concentration to be increased [20,21], and thus cAMP tends to increase [9], this effect is minor and may not be able to attain simultaneous consumption of multiple sugars (data not shown).

## Conclusion

The transcriptional catabolite regulation mechanism was made clear for wild type *E. coli*, and its *crp, mlc, ptsG, pgi*, and *mgsA *gene knockout mutants. The results indicate that catabolite repression can be relaxed, and *crp *as well as *cyaA *transcript levels can be increased by *crp^+^, mgsA, pgi*, and *ptsG *mutants, and thus simultaneous consumption of multiple carbon sources including glucose can be made, whereas the glucose uptake rate became lower as compared to wild type due to inactivation of *ptsG *in all the mutants considered.

## Methods

### Strains used and media composition

The strains used were *E. coli *BW25113 (*F^- ^λ^- ^rph^-1 ^ΔaraBAD_AH33 _lacI^q ^ΔlacZ_WJ16 _rrnB_T14 _ΔrhaBAD_LD78 _hsdR514*) and its single gene knockout mutants lacking *crp *(JW5702) and *mlc *(JW1586), *pgi *(JW3985), *mgsA *(JW5129) and *ptsG *(JW1087), where those were obtained from Keio collection [22]. The *crp *enhanced (*crp^+^*) strain was constructed based on the method of Postai et al. [23] by introducing the mutation responsible for Crp phenotype which lies in the hinge region between the cAMP- and DNA-binding domains. The amino acid substitutions from Gly to Ser at 122 position altered the allosteric nature of Crp, which corresponds to crp*2 in Aiba et al. [24]. All the strains were first precultured in the Luria-Bertani medium. The second preculture and the main culture were carried out using M9 minimal medium containing 5 g/l of glucose for batch culture and 4 g/l for the continuous culture including ^13^C-labeling experiment together with the following components (per liter): 6.81 g Na_2_HPO_4_, 2.99 g KH_2_PO_4_, 0.58 g NaCl and 5.94 g (NH_4_)_2_SO_4_, unless otherwise stated for glucose concentration. The following components were filter sterilized and then added (per liter) with 1 ml of 1 M MgSO_4 _7H_2_O, 1 ml of 0.1 mM CaCl_2 _2H_2_O, 1 ml of 1 mg/L thiamine HCl and 10 ml of trace element solution containing (per liter): 0.55 g CaCl_2 _2H_2_O, 1.67 g FeCl_3 _6H_2_O, 0.1 g MnCl_2 _4H_2_O, 0.17 g ZnCl_2 _2H_2_O, 0.043 g CuCl_2 _2H_2_O, 0.06 g CoCl_2 _2H_2_O, and 0.06 g Na_2_M_O_O_4 _2H_2_O.

### Culture condition

The first precultivation was performed by transferring 0.1 ml glycerol stock to a 10 ml L tube. After 8 h cultivation, the second precultivation was performed by transfering 1 ml of culture broth to a 100 ml T tube. The continuous culture was then conducted in a 2-l fermentor (Venus 25, Biott Co.) with a working volume of 1 l. The pH was controlled at 7.0 ± 0.1 using 2 N HCl or 2 N NaOH, and the temperature was set at 37°C. The air flow rate was 1 vvm (air volume/working volume/min), and the agitation speed was 400 rpm to maintain the dissolved oxygen concentration to be at 35%-40% (v/v) of air saturation [15]. The CO_2 _and O_2 _concentrations were monitored using an off-gas analyzer (BMJ-02 PI, ABLE Co., Japan). The dilution rates in the continuous culture were 0.2, 0.4, 0.6 and 0.7 h^-1 ^for the wild type, and 0.2 h^-1 ^for the mutants. The triplicate samples were collected at the steady state which was confirmed by the constant cell density and CO_2 _concentration in the off-gas. It generally took 3 residence times to achieve the steady state.

### Analytical method

Bacterial growth was monitored by measuring the optical density of the culture broth at 600 nm (OD_600_) using a spectrophotometer (Ubet-30, Jasco, Tokyo, Japan). It was then converted to dry cell weight (DCW) based on the OD_600_-DCW relationship previously obtained [25]. Glucose, xylose and acetate concentrations in the medium were measured using commercially available kits (Wako Co., Osaka, Japan for glucose and xylose; Roche, Molecular Biochemical, Mannheim, Germany for acetate).

### RNA preparation

Total RNA was isolated from *E. coli *cells by Qiagen RNeasy Mini Kit (QIAGEN K.K., Japan) according to the manufacture's recommendation. The quantity and purity of RNA were determined by the optical density measurements at 260 and 280 nm and by 1% formaldehyde agarose gel electrophoresis. The primer sequence for respective genes used in this study were reported elsewhere [19]. Criteria for the design of the gene-specific primer pairs were followed according to [26]. The primers used in this study were synthesized at Hokkaido System Science Co. (Sapporo, Hokkaido, Japan). In all cases, the primer-supplied company confirmed the purity and absolute specificity of the primers.

### cDNA synthesis and PCR amplification

RT-PCR reactions were carried out in a Takara PCR Thermal Cycler (Takara TP240, Japan) using Qiagen One-step RT-PCR Kit (QIAGEN K.K., Japan). The reaction mixture was incubated for 30 min at 50°C for reverse transcription (cDNA synthesis) followed by 15 min incubation at 95°C for initial PCR activation. Then, the process was subjected to 30 cycles of amplification which consisted of a denaturing step (94°C for 1 min), an annealing step (approximately 5°C below melting temperature of primers for 1 min) and an extension step (72°C for 1 min), and finally the reaction mixture was incubated for 10 min at 72°C for final extension. To check for nucleic acid contamination, one negative control was run in every round of RT-PCR. This control lacks the template RNA in order to detect possible contamination of the reaction components. 5 ml of amplified products were run on a 1% agarose gel. Gels were stained with 1 μg/ml of ethidium bromide, photographed using a Digital Image Stocker (DS-30, FAS III, Toyobo, Osaka, Japan) under UV light and analyzed using Gel-Pro Analyzer 3.1(Toyobo, Osaka, Japan) software. Although the PCR products obtained for all the genes showed the predicted sizes on agarose gel, the identity of amplified fragments of some genes was demonstrated by DNA sequencing. In order to determine the optimal amount of input RNA, the two-fold diluted template RNA was amplified in RT-PCR assays under identical reaction conditions to construct a standard curve for each gene product. When the optimal amount of input RNA was determined for each gene product, RT-PCR was carried out under identical reaction conditions to detect differential transcript levels of genes. The gene *dnaA*, which encodes for DnaA, a replication initiation factor in *E. coli *and is not subjected to variable expression, i.e. abundant expression at relatively constant rate in most cells, was used as an internal control for the RT-PCR determinations. To calculate the standard deviation, RT-PCR was independently performed three times for each gene under identical reaction condition. To ensure that the observed changes were statistically significant, the measurements were made in triplicate, and the Student's t-test was applied.

### ^13^C-labeling experiments

The labeling experiments were started after the steady state was confirmed. The unlabeled (naturally labeled) feeding medium was replaced by an identical medium containing 3.2 g/l of unlabeled glucose, 0.4 g/l [U-^13^C] glucose, and 0.4 g/l [1-^13^C] glucose. Biomass samples were taken after another two residence times, where 200 mL of culture broth was taken for the measurement of biomass and extracellular metabolite concentrations, and the remaining 800 mL of the culture broth was processed for gas chromatography-mass spectrometry (GC-MS) analysis. The biomass sample was kept on ice for 2-3 min, and the sample was centrifuged at 6,000 rpm at 2°C for 15 min [27]. The cell pellets were washed three times with 20 mM Tris-HCl at pH 7.6 and suspended in 10 mL of 6 M HCl. The mixture was then hydrolyzed at 105°C for 15 h in a sealed glass tube. During acid hydrolysis, tryptophan and cysteine were oxidized, and glutamine and asparagine were deaminated. The hydrolysate was evaporated to dryness. The dried material was dissolved in Milli-Q water and filtered through a 0.22-μm pore-size filter and evaporated to dryness. About 1.5 ml acetonitrile was added in the dried hydrolyte and incubated at room temperature overnight. After the color of liquid was turned to slight yellow, it was filtrated through 0.22-μm pore-size filter. The filtrate was then derivatized by N-(tert-butyldimethylsilyl)-N-methyl-trifluoroacetamide (Aldrich, USA) at 110°C for 30 min and was transferred to GC-MS sample tube for analysis [27].

### Metabolic flux analysis

Preparation of biomass hydrolysates and recording of the GC-MS spectra (PerkinElmer, Germany) were made as described previously [27,28]. The program Turbomass Gold (PerkinElmer, Germany) was used for peak assignment and MS data processing. The main idea is to perform isotopomer balance on carbon atoms in order to track the fate of the labeled carbon atoms from the substrate [29]. Isotopomer balance enables us to determine the isotopomer distributions of the intracellular metabolites in the central metabolic network. Since the isotopomer distributions of amino acids can be inferred from the isotopomer distributions of their corresponding precursors, the GC-MS signals for the amino acids can then be simulated. A set of flux distributions is then determined by minimizing the differences between the experimental and simulated data. For the estimation of GC-MS signals using isotopomer balance, three types of corrections were made to take into account the effects of natural abundance, non-steady state condition, and skewing effect. First, the isotopomer distributions of the input substrate were corrected for natural abundance in the unlabeled glucose and impurity in the labeled glucose. Second, the isotopic steady-state condition is normally not attained at the time of harvesting biomass. Thus, the simulated data have to be corrected based on the actual harvesting time by assuming first-order washout dynamics. Finally, the simulated GC-MS data were corrected for the natural isotope abundance of O, N, H, Si, S, and C atoms in the derivatizing agent using the correction matrices.

The detailed flux calculation is given previously [27]. Briefly, the best-fit intracellular fluxes were estimated by minimization of the deviation between experimental GC-MS data and the estimated values, using the iterative scheme in the minimization procedure. A set of intracellular fluxes (including net fluxes and exchange fluxes) that gives the minimum deviation could be taken as the best fit. The stage optimization was made by both local search and with global search such as Genetic algorithm [27].

### Estimation of the specific ATP production rate and specific NAPH production rate

The ATP production is made either by substrate level phosphorylation and oxidative phosphorylation, where the reducing power of NADH and FADH_2 _can contribute in generating ATP via oxidative phosphorylation. The pathways involved in electron transport and oxidative phosphorylation have variable stoichiometry due to the use of different dehydrogenases and cytochromes. Then the specific ATP production rate may be estimated by the following equation:(1)

where *OP_NADH _*and *OP*_*FADH*_2 __is the specific ATP production rates via oxidative phosphorylation, and those may be estimated by(1a)

and(1b)

where *(P/O) *and *(P/O)*are the P/O ratios for NADH and FADH_2_, respectively.

Here, we set *(P/O) *to be 2 and *(P/O)*' to be 1.

The specific NADPH production rate *v*_*NADH *_may also be estimated by:(2)

## Abbreviations

### Metabolites

3PG: 3-Phosphoglyceric acid; 6PG: 6-Phosphogluconolactone; AKG: 2-Keto-D-gluconate; AcCoA: Acetyl-CoA; AcP: Acetylphosphate; ASP: Aspartate; ADP: Adenosine diphosphate; ATP: Adenosine-5'-triphosphate; AMP: Adenosine monophosphate; cAMP: Cyclic AMP; CIT: Citrate; DHAP: Dihydroxyacetone phosphate; E4P: Erythrose 4-phosphate; F1,6-BP: Fructose 1,6-bisphosphate; F6P: Fructose 6-phosphate; FUM: Fumarate; G6P: Glucose-6-phosphate; GAP: Glyceraldehyde 3-phosphate; GLX: Glyoxylate; ICIT: Isocitrate; NAD/NADH: Nicotinamide adenine dinucleotide; NADP/NADPH: Nicotinamide adenine dinucleotide phosphate; MAL: Malate; OAA: Oxaloacetate; P: Phosphate; PEP: Phosphoenolpyruvate; PYR: Pyruvate; R5P: Ribulose 5-phosphate; Ru5P: Ribose 5-phosphate; S7P: Sedoheptulose 7-phosphate; SUCCoA: Succinyl-CoA; SUC: Succinate; X5P: Xylulose 5-phosphate.

### Protien (enzyme)

Ack: Acetate kinase; AKGDH: 2-Keto-D-gluconate dehydrogenase; 6PGDH: 6-Phosphogluconate dehydrogenase; EI: Eyme I; EIIA: Ezyme IIA; EIICB: Ezyme IIB; EI-P: Posphorylated state of EI; EIIA-P: Posphorylated state of EIIA; EIICB-P: Posphorylated state of EIICB; G6PDH: Glucose-6-phosphate dehydrogenase; GAPDH: Glyceraldehyde 3-phosphate dehydrogenase; ICDH: Isocitrate dehydrogenase; HPr: Histidine-containing protein; HPr-P: Phorylated state of HPr; Mdh: Malate dehydrogenase; Mez: Malic enzyme; Pck: Phosphoenolpyruvate carboxykinase; PDH: Pyruvate dehydrogenase; Pfk: Phosphofructokinase; Pyk: Pyruvate kinase; SDH: Succinate dehydrogenase; UQ: Ubiquinone;

### Gene

*aceA*: Isocitrate lyase gene; *aceB*: Malate synthase A gene; *aceE, F*: Pyruvate dehydrogenase gene; *ackA*: Acetate kinase gene; *acnA/B*: Aconitase gene; *acs*: Acetyl-coenzyme A synthetase; *arcA/B*: Anoxic redox control protein; *atpA*: ATP synthase; *cra*: Catabolite repressor/activator; *crp*: Cyclic AMP receptor; *crr*: Catabolite repressor; *cyaA*: adenylate cyclase gene; *cyoABCDE*: Cytochrome o oxidase gene; *cydAB*: Cytochrome d oxidase gene; *edd*: 6-Phosphogluconate dehydrogenase gene; *eda*: 2-Keto-3-deoxy-6-phosphogluconate aldolase gene; *fadR*: Fatty acid metabolism regulator; *fnr*: Fumarate/nitrate reduction transcriptional regulator; *fumA, B, C*: Fumarase gene; *fur*: Fe-responsive gene regulator; *gltA*: Citrate synthase gene; *gnd*: 6-phosphogluconate dehydrogenase gene; *icdA*: Isocitrate dehydrogenase A gene; *iclR*: Isocitrate lyase regulator; *lpdA*: Dihydrolipoamide dehydrogenase gene; *mdh*: Malate dehydrogenase gene; *mgsA*: Methylglyoxal synthase gene; *mlc*: DNA-binding transcriptional repressor; *ndh*: NADH dehydrogenase gene; *nuoABCEFGHIJKLMN*: NADH:ubiquinone oxidoreductase; *pckA*: Phosphoenolpyruvate carboxykinase gene; *pfkA*: 6-Phosphofructokinase I gene;

*pgi*: Glucose-6-phosphate isomerase gene; *pgk*: Phosphoglycerate kinase gene; *ppc*: Phosphoenolpyruvate carboxylase gene; *ppsA*: Phosphoenolpyruvate synthase gene; *pta*: Phosphate acetyltransferase gene; *ptsG, ptsHI*: Pts gene; *pykA, F*: Pyruvate kinase gene; *rpoS*: RNA polymerase sigma factor; *sdhC*: Succinate dehydrogenase; *sodA*: Speroxide dismutase; *soxR/S*: Superoxide stress regulon; *sdhABCD*: Succinate dehydrogenase gene; *sucA*: 2-Oxoglutarate decarboxylase; *sucB*: Dihydrolipoamide succinyltransferase; *sucC*: Succinyl-CoA ligase gene; *sucD*: Succinyl-CoA synthetase gene; *talAB*: Transaldolase gene; *tktAB*: Transketolase gene; *tpiA*: Triose phosphate isometase; *ubiE*: Uuinone/menaquinone biosynthesis methyltransferase gene; *yfid*: Autonomous glycyl radical cofactor; *zwf*: Glucose 6-phosphate dehydrogenase gene.

## Competing interests

The authors declare that they have no competing interests.

## Authors' contributions

RY carried out fermentation, GC-MS and RT-PCR experiments, assayed, made statistical analysis, YH carried out fermentation experiments, DS carried out fermentation and GC-MS experiments, KN constructed *crp^+ ^*mutant and prepared other single gene knockout mutants. YQ discussed on the result obtained, and participated in preparing the manuscript, KS considered experimental design, analyzed the result, and prepared manuscript. All authors read and approved the final manuscript.

## Supplementary Material

Additional file 1 **Global regulators and their regulated genes**. Click here for file

Additional file 2 **The effect of dilution rate on the metabolic flux distribution**. All fluxes are given as absolute values (mmol/g/h) and as values relative to the specific glucose consumption rate (in parentheses), values in < > indicate exchange coefficients. Click here for file

Additional file 3 **The effect of dilution rate (specific growth rate) on the specific ATP production rate (3a) and specific NADPH production rate (3b)**. Filled square symbols are from the present study, open triangle symbols are from [30], while open circle symbols from [31]. Click here for file

Additional file 4 **Batch fermentation result of using a mixture of glucose and xylose as a carbon source**. (a) wild type, (b) *crp*^+ ^mutant. Click here for file

Additional file 5 **Continuous fermentation result of using a mixture of glucose and xylose as a carbon source**. (a) wild type, (b) *crp^+ ^*mutant. Click here for file

Additional file 6 **Continuous fermentation result of using a mixture of glucose and xylose as a carbon source for *ptsG *mutant**. Click here for file

Additional file 7 **Batch fermentation result of using glucose as a carbon source for Δ*crp***. Click here for file

Additional file 8 **Batch fermentation result of using glucose as a carbon source for *mlc *mutant**. Click here for file
