# Supplementary material for: Catabolic regulation analysis of Escherichia coli and its crp, mlc, mgsA, pgi and ptsG mutants
Source: Microb Cell Fact. 2011 Aug 11;10:67. doi: 10.1186/1475-2859-10-67 (PMC3169459; doi:10.1186/1475-2859-10-67)
Supplement: Additional file 1 — Global regulators and their regulated genes. [file 1475-2859-10-67-S1.PDF]

Additional file 1 a– Global regulators and their regulated genes

Global Regulators	Regulation	Metabolic Pathway Genes
Crp/Cya	+	*aceEF, acnAB, acs, focA, fumA, fur, gltA, malT, manXYZ, mdh, mlc, pckA, pflB, pgk, ptsG, sdhCDAB, sucABCD*
	–	*cyaA, lpdA, rpoS*
Cra	+	*aceBAK, acnA, cydB, icdA, pckA, pgk, ppsA*
	–	*acnB, adhE, eda, edd, pfkA, pykF, zwf*
ArcA/B	+	*cydAB, focA, pflB*
	–	*aceBAK, aceEF, acnAB, cyoABCDE, fumAC, gltA, icdA, lpdA, mdh, nuoABCEFGHIJKLMN, pdhR, sodA, sdhCDAB, sucABCD*
Fnr	+	*acs, focA, frdABCD, pflB, yfiD*
	–	*acnA, cyoABCDE, cydAB, fumA, fnr, icdA, ndh, nuoABCEFGHIJKLMN, sdhCDAB, sucABCD*
Mlc	–	*crr, ptsG, ptsHI, manXYZ, malT*
FadR	+	*iclR*
	–	

IclR	–	*aceBAK, acs*
SoxR/S	+	*acnA, sodA, zwf, fumC, fur*
	–	
RpoS	+	*acnA, acs, adhE, fumC, gadAB, talA, tktB, poxB, osmC*
	–	*ompF*
Fur	+	
	–	*entABCDE, sodA*
PdhR	+	
	–	*aceEF, lpdA*
