# Supplementary material for: Catabolic regulation analysis of Escherichia coli and its crp, mlc, mgsA, pgi and ptsG mutants
Source: Microb Cell Fact. 2011 Aug 11;10:67. doi: 10.1186/1475-2859-10-67 (PMC3169459; doi:10.1186/1475-2859-10-67)

Additional file 3 – The effect of dilution rate (specific growth rate) on the specific ATP production rate (3a) and specific NADPH production rate (3b)

Filled square symbols are from the present study, open triangle symbols are from [28], while open circle symbols from [29]

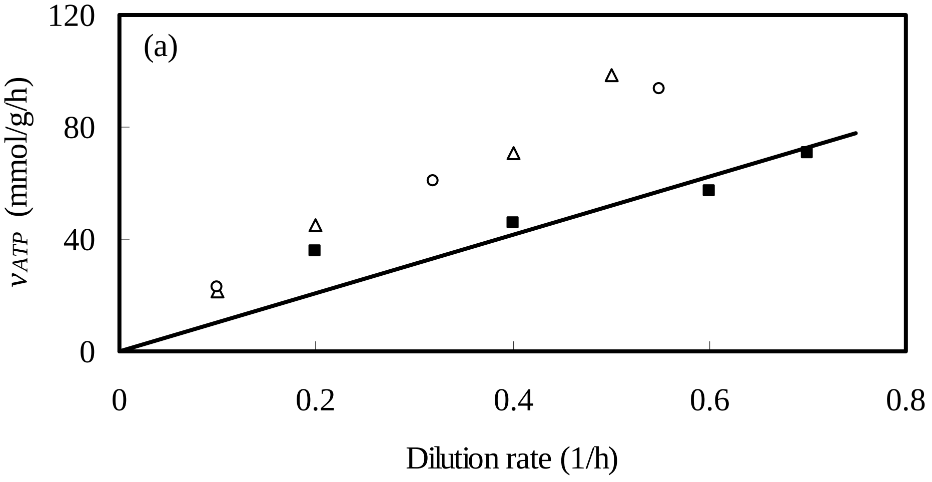

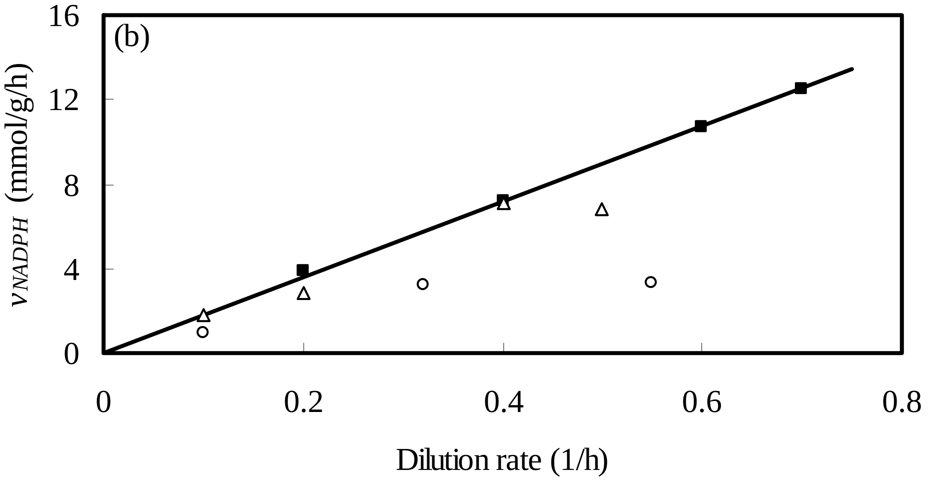

Supplement: Additional file 3 — The effect of dilution rate (specific growth rate) on the specific ATP production rate (3a) and specific NADPH production rate (3b). Filled square symbols are from the present study, open triangle symbols are from [30], while open circle symbols from [31]. [file 1475-2859-10-67-S3.PDF]
