# Supplementary material for: Catabolic regulation analysis of Escherichia coli and its crp, mlc, mgsA, pgi and ptsG mutants
Source: Microb Cell Fact. 2011 Aug 11;10:67. doi: 10.1186/1475-2859-10-67 (PMC3169459; doi:10.1186/1475-2859-10-67)

Additional file 5 – Continuous fermentation result of using a mixture of glucose and xylose as a carbon source. (a) wild type; (b) *crp*$^{+}$ mutant

(a)

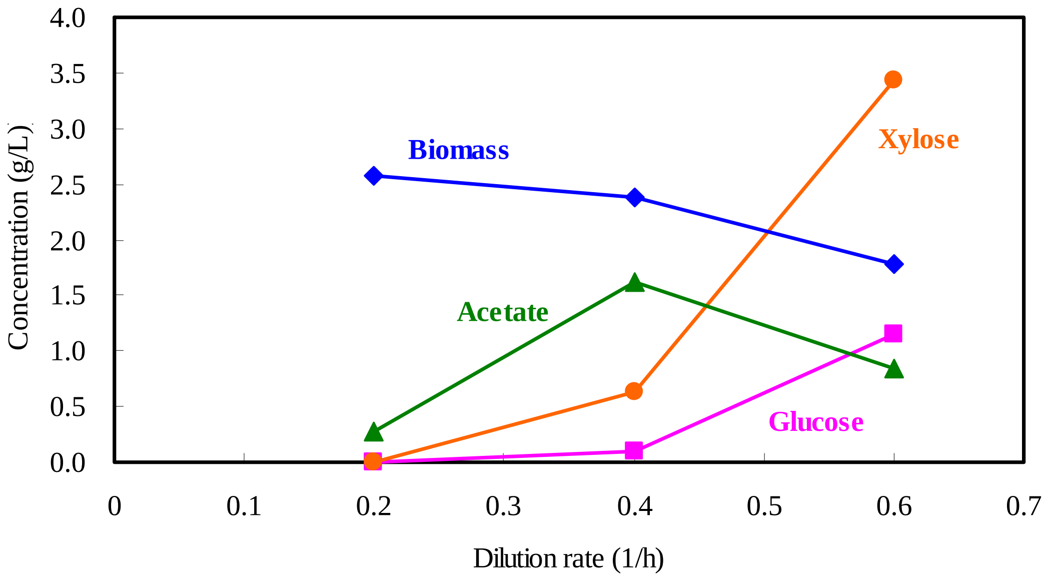

(b)

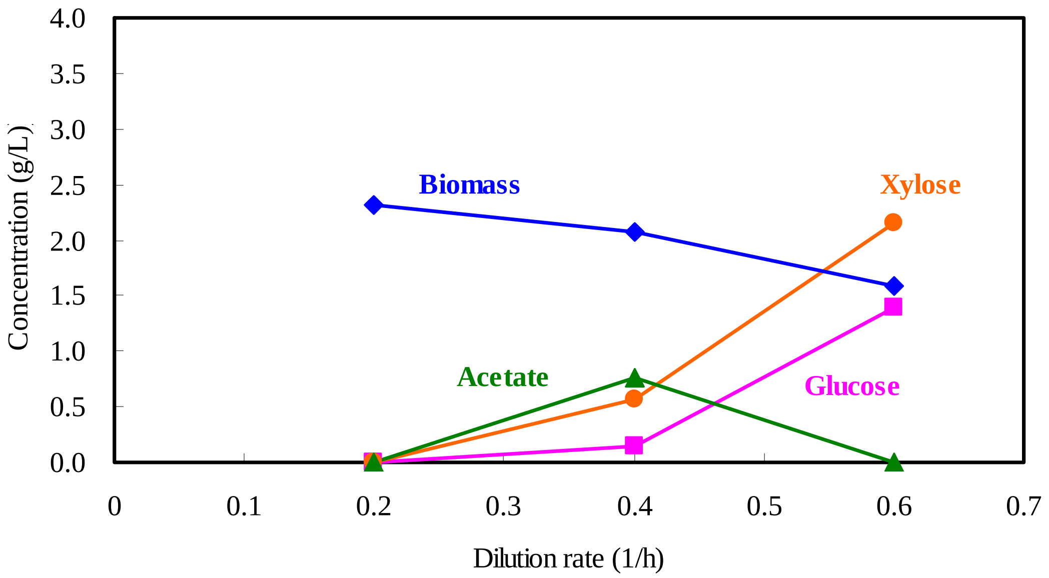

Supplement: Additional file 5 — Continuous fermentation result of using a mixture of glucose and xylose as a carbon source. (a) wild type, (b) crp+ mutant. [file 1475-2859-10-67-S5.PDF]
