# Supplementary material for: Catabolic regulation analysis of Escherichia coli and its crp, mlc, mgsA, pgi and ptsG mutants
Source: Microb Cell Fact. 2011 Aug 11;10:67. doi: 10.1186/1475-2859-10-67 (PMC3169459; doi:10.1186/1475-2859-10-67)

Additional file 6 – Continuous fermentation result of using a mixture of glucose and xylose as a carbon source for *ptsG* mutant

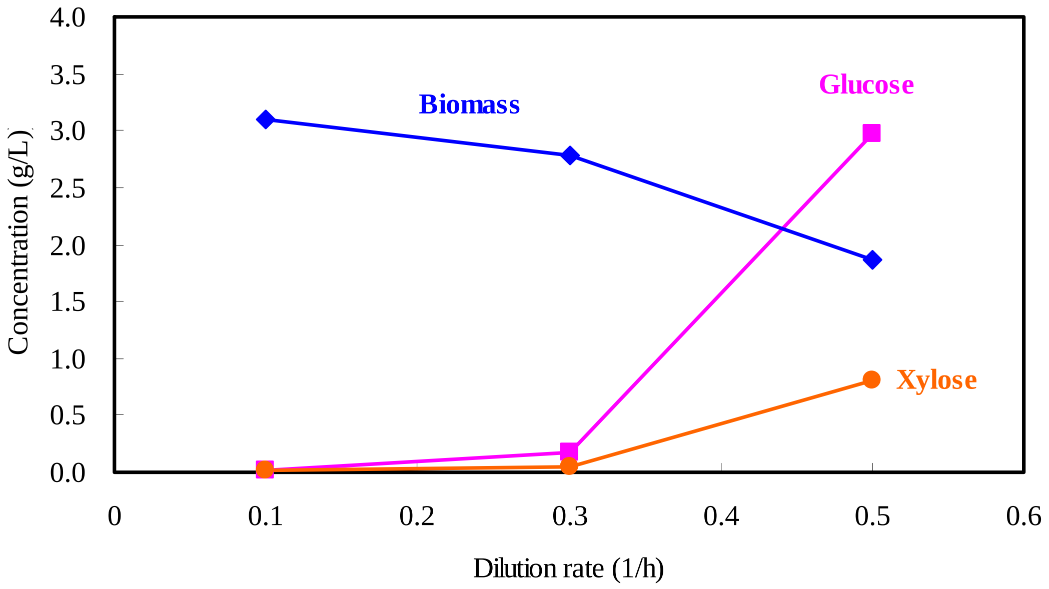

Supplement: Additional file 6 — Continuous fermentation result of using a mixture of glucose and xylose as a carbon source for ptsG mutant. [file 1475-2859-10-67-S6.PDF]
