# Supplementary figures and images for: Catabolic regulation analysis of Escherichia coli and its crp, mlc, mgsA, pgi and ptsG mutants
Source: Microb Cell Fact. 2011 Aug 11;10:67. doi: 10.1186/1475-2859-10-67 (PMC3169459; doi:10.1186/1475-2859-10-67)

**Additional file 7 – Batch fermentation result of using glucose as a carbon source for  $\Delta crp$**

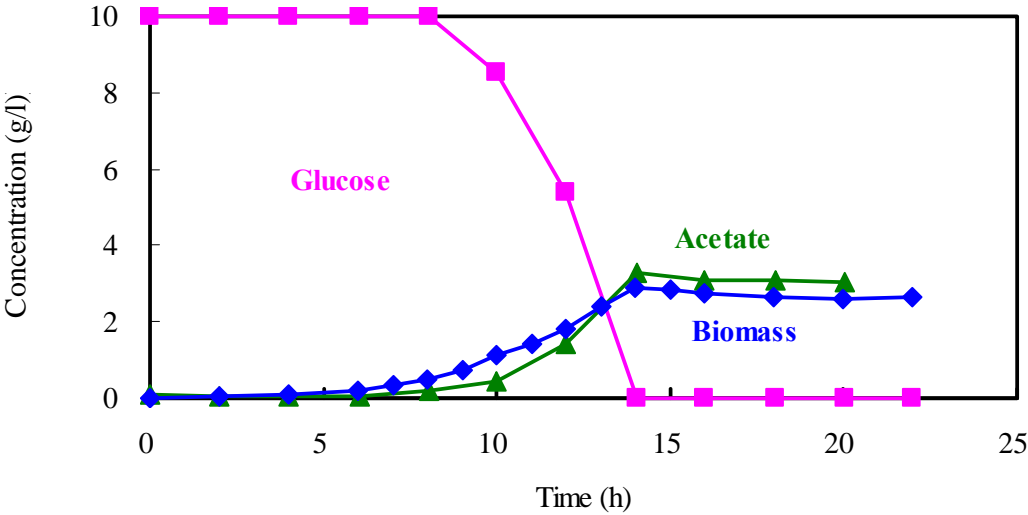

Supplement: Additional file 7 — Batch fermentation result of using glucose as a carbon source for Δcrp. [file 1475-2859-10-67-S7.PDF]

**Additional file 8 – Batch fermentation result of using glucose as a carbon source for *mlc* mutant**

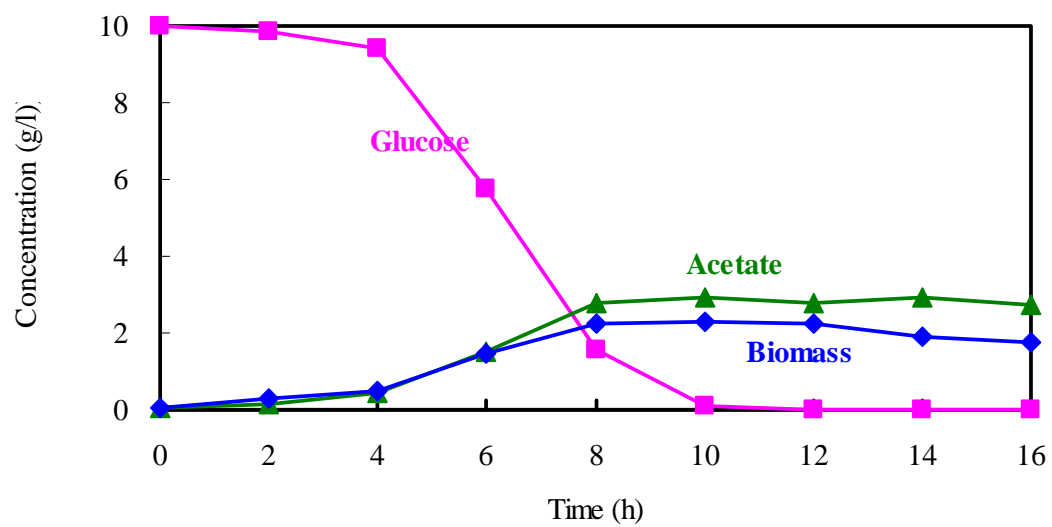

Supplement: Additional file 8 — Batch fermentation result of using glucose as a carbon source for mlc mutant. [file 1475-2859-10-67-S8.PDF]
