# Supplementary material for: Catabolic regulation analysis of Escherichia coli and its crp, mlc, mgsA, pgi and ptsG mutants
Source: Microb Cell Fact. 2011 Aug 11;10:67. doi: 10.1186/1475-2859-10-67 (PMC3169459; doi:10.1186/1475-2859-10-67)
Supplement: Supplementary file 7 [file 1475-2859-10-67-S2.DOC]

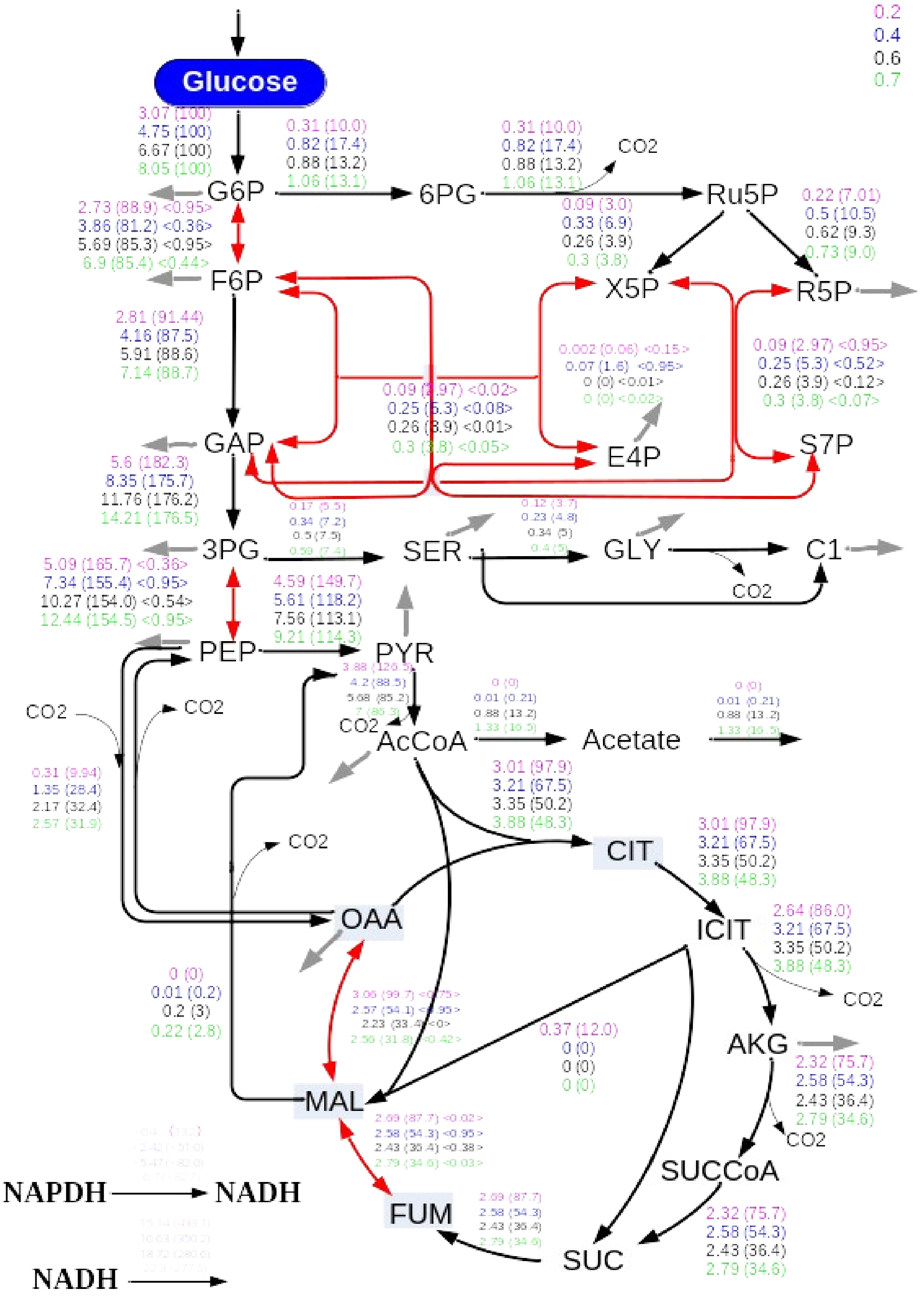
0.2
0.4
0.6
0.7
Glucose
3.07 (100)
4.75 (100)
6.67 (100)
8.05 (100)
G6P
0.31 (10.0)
0.82 (17.4)
0.88 (13.2)
1.06 (13.1)
6PG
CO2
Ru5P
2.73 (88.9) <0.95>
3.86 (81.2) <0.36>
5.69 (85.3) <0.95>
6.9 (85.4) <0.44>
F6P
0.09 (3.0)
0.33 (6.9)
0.26 (3.9)
0.3 (3.8)
0.22 (7.01)
0.5 (10.5)
0.62 (9.3)
0.73 (9.0)
X5P
R5P
2.81 (91.44)
4.16 (87.5)
5.91 (88.6)
7.14 (88.7)
0.002 (0.06) <0.15>
0.07 (1.6) <0.95>
0 (0) <0.01>
0 (0) <0.02>
0.09 (2.97) <0.95>
0.25 (5.3) <0.52>
0.26 (3.9) <0.12>
0.3 (3.8) <0.07>
0.09 (2.97) <0.02>
0.25 (5.3) <0.08>
0.26 (3.9) <0.01>
0.3 (3.8) <0.05>
GAP
E4P
S7P
5.6 (182.3)
8.35 (175.7)
11.76 (176.2)
14.21 (176.5)
3PG
SER
GLY
C1
CO2
5.09 (165.7) <0.36>
7.34 (155.4) <0.95>
10.27 (154.0) <0.54>
12.44 (154.5) <0.95>
4.59 (149.7)
5.61 (118.2)
7.56 (113.1)
9.21 (114.3)
PEP
PYR
CO2
CO2
CO2
AcCoA
Acetate
0.31 (9.94)
1.35 (28.4)
2.17 (32.4)
2.57 (31.9)
3.01 (97.9)
3.21 (67.5)
3.35 (50.2)
3.88 (48.3)
CO2
CIT
3.01 (97.9)
3.21 (67.5)
3.35 (50.2)
3.88 (48.3)
OAA
ICIT
2.64 (86.0)
3.21 (67.5)
3.35 (50.2)
3.88 (48.3)
0 (0)
0.01 (0.2)
0.2 (3)
0.22 (2.8)
CO2
0.37 (12.0)
0 (0)
0 (0)
0 (0)
AKG
2.32 (75.7)
2.58 (54.3)
2.43 (36.4)
2.79 (34.6)
MAL
CO2
SUCCoA
NAPDH
NADH
FUM
2.32 (75.7)
2.58 (54.3)
2.43 (36.4)
2.79 (34.6)
SUC
NADH
